# The C-shaped Canal System in Mandibular Molars of a Saudi Arabian Population: Prevalence and Root Canal Configurations Using Cone-Beam Computed Tomography

**DOI:** 10.7759/cureus.25343

**Published:** 2022-05-26

**Authors:** Yousef Alnowailaty, Faisal Alghamdi

**Affiliations:** 1 Conservative Dentistry, King Abdulaziz University, Jeddah, SAU; 2 Oral Biology, King Abdulaziz University, Jeddah, SAU

**Keywords:** saudi population, longitudinal groove, c-shaped canal, mandibular molar, cone-beam computed tomography

## Abstract

Objectives: This retrospective study aimed to assess the prevalence of the C-shaped canal system in mandibular first and second molars in a Saudi Arabian population using cone-beam computed tomography (CBCT).

Materials and Methods: The sample included 300 CBCT images from adults aged 18 to 80 years (38.7 ± 17.9) (150 females and 150 males). All images were analyzed to detect the C-shaped root canal according to Fan’s criteria. We also categorized our findings according to the gender and direction of the longitudinal groove. Data were reported as frequencies and percentages. The Chi-squared test was used to analyze proportional differences, with the significance level set at ≤0.05.

Results: The C-shaped canals were observed in 146 mandibular first molars (24.33%) and 182 second molars (30.33%). Male patients had a significantly higher prevalence of C-shaped canals in mandibular second molars than females (P=0.003). The longitudinal groove was most typically detected on the root's lingual surface (53.35%).

Conclusion: The incidence of the C-shaped canal in a Saudi Arabian population was 27.33% in the mandibular molars. The longitudinal groove was mostly on the lingual surface. Males showed a higher prevalence of the C-shaped canal than females.

## Introduction

There are many anatomical variations in the root canal system. Some variations can significantly increase the difficulty of endodontic treatment [1]. Knowledge of the anatomical complexity of the root canal system and its potential to vary can enhance the effective cleaning and shaping of the pulp canal space, thereby improving treatment efficiency and the long-term prognosis of the endodontic treatment [1,2]. One of the most challenging anatomical complexities of the root canal system is the C-shaped canal [3,4].

In 1979, Cooke & Cox described the first anatomical variety of the C-shaped canal system [5]. On the axial level, the root canal orifice resembles a C and is often referred to as "C-shaped" because the fin or web connects the separate root canals [6].

The orifice of each canal is located below the cement-enamel junction (CEJ). The orifice is single ribbon-shaped, with a 180 ° arc connecting all major channels or an arc connecting one of the mesial channels (buccal or lingual) to the distal channel [6,7]. The C-shaped canal is hypothesized to be formed by damages to the root sheath of the Hertwig epithelium on the lingual or buccal side of the root surface during development [8].

In 1991, Melton et al. developed a classification for the C-shaped channels [9]. Fan et al. [10] further modified Melton’s classification as follows - (i) C1: Continuous “C” with no division or separation, (ii) C2: Semi‑colon canal outline due to an interruption in the continuity of “C” outline with no less than <60° of either “α” or “β” angle, (iii) C3: 2(d) or 3(c) separate canals with both “α” and “β” angles <60°, (iv) C4: One oval or round canal in horizontal cross‑section, and (v) C5: No canal lumen, usually noted close to the apex.

Shortly after computed tomography (CT) was applied in endodontics [11], cone-beam computed tomography (CBCT) was introduced to dentistry in the end nineties. The CBCT provides numerous advantages over traditional CT, such as higher image resolution, reduced scanning time, and lower radiation doses [12,13]. The increased popularity of CBCT in dentistry is due to the high degree of accuracy it offers in comparison with conventional periapical radiographs. This is especially beneficial for assessing the radiographic changes associated with a possible medical condition or the presence of different morphological changes [14].

The prevalence of C-shaped canals and their different configurations in mandibular first and second molars have been evaluated in different populations [15-18]. However, only three studies have been conducted in Saudi Arabia using CBCT or periapical radiographs to evaluate the prevalence of C-shaped canals in mandibular molars [1,19,20]. These studies are designed to enhance the clinician’s knowledge in understanding and predicting root canal geometry before beginning endodontic therapy. In addition, the prevalence of C-shaped canals and their impact on the Saudi Arabian population have yet to be adequately discussed in the literature. This study aimed to investigate the prevalence of C-shaped root canals, the anatomic longitudinal groove, and gender distributions in the mandibular first molar (1stM) and second molar (2ndM) in a Saudi population using CBCT.

## Materials and methods

Sample selection

The study was performed at the King Abdulaziz University dental hospital, oral and maxillofacial radiology department. Random screening of 2946 CBCT scans was conducted based on inclusion and exclusion criteria. Only Saudi Arabian citizens who are residents of Jeddah City, with high-quality CBCT scans, and at least one mandibular 1stM or 2ndM were included in this study. A scan was excluded if it was not of a Saudi citizen, if the image was unclear/distorted, or if at least one mandibular 1stM or 2ndM is not detected.

A multi-stage stratified random sample with two database groups for CBCT scans was used in this investigation. The screening process resulted in 300 CBCT scans from 150 males and 150 females, aged 18 to 80 years (mean ± standard deviation [SD]=38.7 ± 17.9). Images from each database group were captured between January 2013 and December 2021. A CBCT was requested due to different reasons, including preoperative screening for implant placement, impacted tooth extraction, orthodontic treatment, facial injury, and before root canal therapy (RCT) in teeth with suspected complex root canal anatomy. The scans provided demographic information such as gender, country, and age, but no additional patient identifying information was given. To rule out the effects of ethnic differences on the type or number of mandibular 1stM and 2ndM with C-shaped canals, all study participants lived in Jeddah and only Saudi Arabian citizens were included. Due to the lack of available datasets, all Saudi citizens and residents who did not live in Jeddah or who belonged to different provinces of Saudi Arabia were excluded from this study. The Research Ethics Committee of the Faculty of Dentistry granted ethical approval for this study (Proposal No.: 353-12-21). All participants signed an informed consent form acknowledging that their data would be used anonymously for research purposes. This study was conducted as per the Helsinki Declaration.

Power analysis for sample size

An independent t-test was used to calculate the power of this study. Based on values from the t-test using an alpha (α) level of 0.05 (5%), the power was 0.92 for a total of 1000 patients (500 per group for both genders). Sample size estimation was calculated by using Power and Sample Size Calculation version 3.1.6 (PS software, Vanderbilt University, Nashville, TN, USA).

Image acquisition

An i-CAT 1719 3D digital imaging system (Imaging Sciences International, Hatfield, PA, USA) was used for CBCT image acquisition. The following standardized protocol and settings were applied for the included recorded scans: i) exposure at 120kV and 5-8mA, ii) exposure duration of 17.5-26.9 seconds, and iii) CBCT scans set at 8×8 cm field of view (FOV) and a voxel size of 0.125mm.

Image evaluation

For image reconstruction and measurements, the OnDemand 3D Imaging Software (Cybermed, Seoul, South Korea) was used. The prevalence of C-shaped root canal systems as classified by Fan et al. [10] in the mandibular 1stM and 2ndM was analyzed. The cross-sections of each C-shaped canal was analyzed at three levels - (a) coronal: under the canal orifice by 2mm, (b) middle: the root length from canal orifice to radiographic apex divided by two, and (c) apical: relative to the radiographic apex by 2 mm. All detected C-shaped root canals were categorized based on Fan’s classification [10]. Furthermore, the location of the longitudinal groove of the root (lingual vs. buccal) was evaluated. Scans were reconstructed in all planes (coronal, axial, and sagittal planes). Moreover, multiplanar reconstruction (MPR) was conducted to capture the root canal system in detail. The MPR was performed in the coronal-apical, and then apical-coronal directions. In instances when the scan was unclear, the procedure was repeated. Thus, each tooth was inspected three-dimensionally.

The authors examined the scans of the 1stM and 2ndM to identify the C-shaped canals and classify them using Vision software (Imaging Science International, Hatfield, PA, USA). Before the actual sample assessments, the calibration process of the two examiners and one consultant radiologist with endodontic experience was performed by reviewing 100 randomly selected CBCT images that were not included in the study. Moreover, the process was repeated at two different time points during the investigation, at two weekly intervals. The radiologist ensured that the CBCT scan assessment was standardized.

Statistical analysis

Collected data were analyzed using Statistical Package for the Social Sciences (SPSS) version 20.0 for Windows (IBM Corp., Armonk, NY, USA). Inter-examiner and intra-examiner reliability for CBCT scan interpretation was done using Cohen's Kappa test. The frequency and percentage of C-shaped canals and their correlations with gender and longitudinal groove location were measured using the Chi-squared test. The statistical level of significance was set at 0.05.

## Results

Inter- and intra-examiner reliability

The inter-examiner agreement for the identification of C-shaped canals (Kappa≥0.998), classification of the C-shaped canals (Kappa≥0.994), and the longitudinal groove location of the C-shaped canals (Kappa≥0.996) was satisfactory.

The intra-examiner reliability for the first examiner was excellent (identification of C-shaped canals Kappa≥0.98, classification of the C-shaped canals Kappa≥0.96, and the longitudinal groove location of the C-shaped canals Kappa≥0.98). Similarly, the second examiner demonstrated excellent intra-examiner reliability scores for the three parameters (Kappa≥0.98, Kappa≥0.95, and Kappa≥0.97).

Prevalence of the C-shaped canal anatomy

In total, 1200 mandibular 1stM (n=600) and 2ndM (n=600) from 300 CBCT images were screened. The overall prevalence of the C-shaped canal in the mandibular 1stM and 2ndM was 27.33% (328, P=0.087) (Table 1). The prevalence of the C-shaped canal in the mandibular 1stM (P=0.196) was less than in mandibular 2ndM (P=0.003) (146 vs.182) (Table 1).

There was a statistically significant association between the C-shaped canal and gender in the mandibular 2ndM (P=0.003), with a slightly higher prevalence among males (35.67%) compared to females (25%). The prevalence of the C-shaped canal in the mandibular 1stM in females (26%) was slightly higher than males (22.67%) but this was not statistically significant (P=0.196). In general, the prevalence of the C-shaped canal in the mandibular molars had a general male predilection (males 29.17% vs. females 25.5%) (Table 1).

**Table 1:** Prevalence of mandibular C-shaped first and second molars root canal configurations according to gender

Mandibular C-shaped molars		Males (%)	Females (%)	Total (%)	Chi-square	P-value
All Mandibular Molars (n=1200)	Absent	425 (70.83%)	447 (74.5%)	872 (72.67%)	2.031	0.087
	Present	175 (29.17%)	153 (25.5%)	328 (27.33%)		
	Total	600 (100%)	600 (100%)	1200 (100%)		
1^st^ Mandibular Molars (n=600)	Absent	232 (77.33%)	222 (74%)	454 (75.67%)	0.905	0.196
	Present	68 (22.67%)	78 (26%)	146 (24.33%)		
	Total	300 (100%)	300 (100%)	600 (100%)		
2^nd^ Mandibular Molars (n=600)	Absent	193 (64.33%)	225 (75%)	418 (69.67%)	8.076	0.003
	Present	107 (35.67%)	75 (25%)	182 (30.33%)		
	Total	300 (100%)	300 (100%)	600 (100%)		

Prevalence of the C-shaped canal configuration types

**Figure 1:**
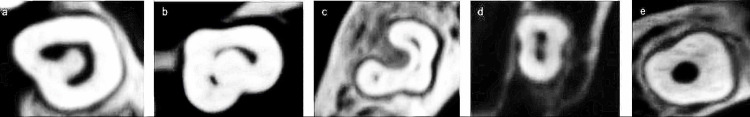
Cone-beam computed tomography axial images of mandibular molars with C-shaped canals. Images show different cross-sections of C-shaped canal configuration classifications (a-e). a: C1, b: C2, c: C3(c), d: C3(d), e: C4

In classifying the C-shaped canal configuration types at all three cross-sectional levels (coronal, middle, and apical) for the mandibular 1stM and 2ndM according to Fan’s classification, there was no statistical significance observed in the prevalence of any one specific type of C‑shaped canal configuration (P=0.524, Tables 2-4). All C-shaped canal configuration types, except for C5, were detected in the mandibular 1stM and 2ndM at all three cross-section levels (Figure 1).

The most observed C‑shaped canal configuration type at the coronal level was C1 (60.67%). The distribution of C1 in the mandibular 1stM (70.55%) was higher than the mandibular 2ndM (52.75%) (P=0.001) (Table 2). The distribution of other canal configuration types in the mandibular 1stM and 2ndM were C2 (14.94%, P=0.000), C3(d) (11.89%, P=0.000) and C4 (10.98%, P=0.000), while the lowest prevalence was C3(c) (1.52%, P=0.603) at the coronal level (Table 2).

**Table 2:** The distribution of categories of C-shaped canal configuration at the coronal level

Coronal Level		Number of Teeth	Chi-square	p-value
C1	1st Molar (n=146)	103 (70.55%)	10.758	0.001
	2nd Molar (n=182)	96 (52.75%)		
	Total (n=328)	199 (60.67%)		
C2	1st Molar (n=146)	4 (2.74%)	30.816	0.000
	2nd Molar (n=182)	45 (24.73%)		
	Total (n=328)	49 (14.94%)		
C3c	1st Molar (n=146)	2 (1.37%)	0.042	0.603
	2nd Molar (n=182)	3 (1.65%)		
	Total (n=328)	5 (1.52%)		
C3d	1st Molar (n=146)	1 (0.68%)	31.535	0.000
	2nd Molar (n=182)	38 (20.88%)		
	Total (n=328)	39 (11.89%)		
C4	1st Molar (n=146)	36 (24.66%)	50.409	0.000
	2nd Molar (n=182)	0 (0.00%)		
	Total (n=328)	36 (10.98%)		
Total	All the molars in coronal level - Total (n=328)	328 (100%)	0.000	0.524

The most observed C‑shaped canal configuration type at the middle level was C2 with (29.88%, P=0.000) (Table 3). The distribution of other canal configuration types in the mandibular 1stM and 2ndM were C3(d) (28.96%, P=0.000), C3(c) (27.74%, P=0.000), and C1 (13.41%, P=0.000). The C4 configuration type was not observed in the middle level (Table 3).

**Table 3:** The distribution of categories of C-shaped canal configuration at the middle level

Middle Level		Number of Teeth	Chi-square	p-value
C1	1st Molar (n=146)	44 (30.14%)	63.347	0.000
	2nd Molar (n=182)	0 (0.00%)		
	Total (n=328)	44 (13.41%)		
C2	1st Molar (n=146)	98 (67.12%)	174.217	0.000
	2nd Molar (n=182)	0 (0.00%)		
	Total (n=328)	98 (29.88%)		
C3c	1st Molar (n=146)	4 (2.74%)	82.061	0.000
	2nd Molar (n=182)	87 (47.80%)		
	Total (n=328)	91 (27.74%)		
C3d	1st Molar (n=146)	0 (0.00%)	107.281	0.000
	2nd Molar (n=182)	95 (52.20%)		
	Total (n=328)	95 (28.96%)		
C4	1st Molar (n=146)	0 (0.00%)	-	-
	2nd Molar (n=182)	0 (0.00%)		
	Total (n=328)	0 (0.00%)		
Total	All the molars in coronal level - Total (n=328)	328 (100%)	0.000	0.524

At the apical level, the most observed C‑shaped canal configuration type was C3(c) (34.45%). The distribution of C3(c) in the mandibular 1stM (45.89%) was significantly higher than the mandibular 2ndM (25.27%, P=0.000) (Table 4). The prevalence of other canal configuration types in the mandibular 1stM and 2ndM was: C3(d), 27.74% (P=0.000), C1, 22.56% (P=0.000), and C4, 13.72% (P=0.000). Moreover, the lowest prevalence was C2 (1.52%, P=0.017) (Table 4).

**Table 4:** The distribution of categories of C-shaped canal configuration at the apical level

Apical Level		Number of Teeth	Chi-square	p-value
C1	1st Molar (n=146)	74 (50.68%)	119.122	0.000
	2nd Molar (n=182)	0 (0.00%)		
	Total (n=328)	74 (22.56%)		
C2	1st Molar (n=146)	5 (3.42%)	6.329	0.017
	2nd Molar (n=182)	0 (0.00%)		
	Total (n=328)	5 (1.52%)		
C3c	1st Molar (n=146)	67 (45.89%)	15.247	0.000
	2nd Molar (n=182)	46 (25.27%)		
	Total (n=328)	113 (34.45%)		
C3d	1st Molar (n=146)	0 (0.00%)	101.030	0.000
	2nd Molar (n=182)	91 (50%)		
	Total (n=328)	91 (27.74%)		
C4	1st Molar (n=146)	0 (0.00%)	41.839	0.000
	2nd Molar (n=182)	45 (24.73%)		
	Total (n=328)	45(13.72%)		
Total	All the molars in coronal level - Total (n=328)	328 (100%)	0.000	0.524

Prevalence of the C-shaped canal in mandibular molars concerning the longitudinal groove location

In mandibular 1stM and 2ndM with a C-shaped canal, the longitudinal groove was most commonly detected on the lingual side (52.74%) followed by buccal and lingual sides collectively (5.18%). The buccally located longitudinal groove was observed in 136 teeth (41.46%, P=0.036), which was statistically significant. Of these, 47.26% was detected in 1stMs and 36.46% in 2ndMs. In contrast, there was no statistical significance relating to the lingually located longitudinal groove detected in 175 teeth (53.35%, P=0.465). Of these, 52.74% was detected in 1stMs and 53.85% in 2ndMs. Collectively, the buccal and lingual longitudinal grooves were observed with statistical significance in 17 teeth (5.18%, P=0.000) (Table 5).

**Table 5:** Distribution of the C-shaped mandibular molars with longitudinal groove location

Type of groove	Mandibular C-shaped molars	Groove Location (%)	Chi-square	P-value
Buccal groove	1^st^ Mandibular C-shaped Molars (n=146)	69 (47.26%)	3.643	0.036
	2^nd^ Mandibular C-shaped Molars (n=182)	67 (36.46%)		
	All Mandibular C-shaped Molars (n=328)	136 (41.46%)		
Lingual groove	1^st^ Mandibular C-shaped Molars (n=146)	77 (52.74%)	0.040	0.465
	2^nd^ Mandibular C-shaped Molars (n=182)	98 (53.85%)		
	All Mandibular C-shaped Molars (n=328)	175 (53.35%)		
Buccal and Lingual grooves	1^st^ Mandibular C-shaped Molars (n=146)	0 (0.00%)	14.838	0.000
	2^nd^ Mandibular C-shaped Molars (n=182)	17 (9.34%)		
	All Mandibular C-shaped Molars (n=328)	17 (5.18%)		

## Discussion

In this retrospective cross-sectional study, the prevalence of the C-shaped canal was assessed in the mandibular 1stM and 2ndM using CBCT scans from citizens of Jeddah city in Saudi Arabia. We presented the prevalence of the C-shaped canal, canal configuration types, and longitudinal groove location at a specific period.

Micro-CT is the most modern and noninvasive ex vivo technique is currently used to assess tooth morphology. This technique provides researchers with more detailed anatomical information than conventional techniques [21]. This ex vivo approach was selected to assess the C-shaped canal morphology in mandibular molars [10]. High-resolution micro-CT scans aided in the accurate visualization and classification of C-shaped canals [10]. Some uncertainties remained, as previous studies had a small sample size, absence of gender information, and/or a lack of accurate position of the tooth relative to the jaw. The CBCT is an in vivo valid approach for evaluating tooth anatomy and morphology that provides detailed information [22]. It is commonly utilized in endodontics and for prevalence investigations, using specific voxel sizes recommended for molars with C-shaped canals, which is compatible with our study findings [23].

The mandibular 1stM generally has a low incidence of the C-shaped canal [24] compared to the mandibular 2ndM [25]. Moreover, frequent variations are noted in the root morphology and canal configuration of the mandibular 2ndM [26]. These findings concurred with our study results that the prevalence of the C-shaped canal in the mandibular 2ndM (30.33%) was higher than the mandibular 1stM (24.33%).

In this study, the incidence of a C-shaped canal in the mandibular 1stM was 24.33%. This incidence is considered high, and contradicted the outcomes previously reported for Saudi 0.19% [1], Portuguese (0.6%) [2], and Brazilian (1.7%) populations [18].

Multiple studies have reported the incidence of the C-shaped root canal in mandibular 2ndM as ranging between 2.7% and 44.5%, depending on the research design and the ethnic group studied [27]. East Asian studies have the highest prevalence of the C-shaped canal, particularly in Korea (40% to 44.5%) [15,28] and China 29% to 39%) [7,29]. In Southern Asia, the Burmese population had the greatest incidence (22.4%) [30], followed by Indians (12.3%) [23] and Thais (10%) [31]. Previous European studies reported a frequency of 8.5% and 14% in Portuguese [2] and Russian populations [32], respectively. In contrast, a Greek investigation involving radiographic interpretation revealed a significantly lower incidence (5%) [33]. The lower percentages could be attributed to the ethnicity of the sample population or a restriction of the radiography approach [33]. The frequency was 14.2 % in a Mexican study [23]. In Brazilian communities, the frequency varied between 3.5% and 15.3% [18, 34]. In the Middle East, prevalence between 8.9% and 21.4% have been reported [16,17,35-39].

Only three previous studies have investigated the C-shaped root canal morphology in a Saudi population [1,19,20]. The finding of these studies showed a lower prevalence of the C-shaped canal in the mandibular 2ndM (7.9%-10.6%) compared to our finding (30.33%). The difference may be attributed to the use of CBCT as an analytical tool in our study, which allowed for a greater sample size with additional data, and we also assessed the canal configuration at different levels of the root and relative to the longitudinal groove [1].

In the current study, at the coronal level of the root, the C1 canal configuration was reported to be the most prevalent. This is consistent with the findings of Mashyakhy et al. [19], and Sönmez Kaplan et al. [39]. In consensus with Sönmez Kaplan et al. [39], the C2 canal configuration was reported to be the most prevalent at the middle level of the root. In contrast, the C3 canal configuration (C3(c) or C3(d)) was reported to be the most prevalent at the apical level of the root. This concurred with the findings of Alfawaz et al. [1], Martins et al. [2], and Mashyakhy et al. [19].

Several investigations have analyzed the relationship between gender and the occurrence of the C-shaped canal. While some studies [29,35] have found no association, our findings are consistent with other investigations [2,19,39] that reported a gender predilection for the C-shaped canal, particularly for females. Interestingly, our results determined a predilection for males.

Furthermore, knowing the location of the longitudinal groove is critical to prevent strip perforation during endodontic therapy [28]. Fan et al. [10] documented the closeness of the isthmus joining the major root canals of the C-shaped geometries and the exterior groove of the root in a microCT (µ-CT) investigation, in which a limited number of the isthmus may be detected relatively close to the groove. In our study, the groove was more commonly observed on the lingual root surface (53.35%), which is consistent with the findings of Alfawaz et al. [1], Martins et al. [2], and Helvacioglu et al. [35].

Study strengths and limitations

Our study evaluated all available CBCT images for the C-shaped canal in the 1stM and 2ndM that met the inclusion and exclusion criteria. This is the first retrospective cross-sectional study that investigated the prevalence of the C-shaped canal in 1stM and 2ndM among the Saudi population in Jeddah city. We examined 300 CBCT scans collected between 2013 and 2021. As it is preferable to avoid exposing patients to radiation to identify C-shaped canal systems, we used CBCT scans that were ordered for other medical/dental reasons. Furthermore, the CBCT scans were obtained from a single hospital. Therefore, possible biases, such as variances in exposure duration and setting, were avoided. One limitation of this study was the attempt to analyze the C-shaped canal 1stM and 2ndM in a small number of villages around the dental hospital. The patients come from various locations, but they were not representative of the entire Saudi population.

## Conclusions

From the current retrospective study, it was concluded that the use of CBCT assessment increasingly helps identify the prevalence of C-shaped canals in mandibular molars in the Saudi Arabian population. The incidence of C-shaped was high in males than females and 2ndM than 1stM. Having a prior knowledge of this anatomic variation helps a clinician to locate and treat these teeth. Furthermore, it's important to consider the CBCT tool for the clinical assessment of C-shaped canal for best results in treatment of mandibular molars. Future clinical and radiographic studies are warranted to validate our findings and provide a comprehensive evaluation and diagnosis of the C-shaped canal in the 1stM and 2ndM.
